# Plastic debris dataset on the Seine river banks: Plastic pellets, unidentified plastic fragments and plastic sticks are the Top 3 items in a historical accumulation of plastics

**DOI:** 10.1016/j.dib.2019.01.045

**Published:** 2019-01-22

**Authors:** Romain Tramoy, Laurent Colasse, Johnny Gasperi, Bruno Tassin

**Affiliations:** a LEESU (UMR MA 102, University of Paris-Est, AgroParisTech), University of Paris-Est Créteil, 61 avenue du Général de Gaulle, 94010 Créteil Cedex, France; b SOS Mal de Seine NGO, France

## Abstract

Plastic pollution in oceans and rivers is of high concern because of its persistence in the environment and its potential impact on ecosystems. However, there is a specific lack of data in rivers. Here we present data from the Seine river banks in a historical polluted shore. Data were classified using international MSFD and OSPAR classifications. The sampled site is a quadrat of 1 m^2^ located downstream in the estuary in a visual maximum along a 1 km shore covered by plastics. A total of 20,259 plastic debris were individually counted, classified and weighted by category for a total mass higher than 4 kg. Half of the plastic debris in number are represented by preproduction pellets.

**Specifications table**

**Table:** 

Subject area	*Environment*
More specific subject area	*Plastic pollution*
Type of data	*Table, figure*
How data was acquired	*Hand collection, visual and chemical identification*
Data format	*analyzed*
Experimental factors	*Air-dried and sorted with the naked eye*
Experimental features	*Sampling and sorting*
Data source location	*Seine estuary, Petiville, France,* Lat. 49.4339; Long. 0.6160
Data accessibility	*Data in this article*
Related research article	A. Bruge C. Barreau, J. Carlot, H. Collin, C. Moreno and P. Maison, Monitoring Litter Inputs from the Adour River (Southwest France) to the Marine Environment, J. Mar. Sci. Eng., 6 (2018) 24–36. 10.3390/jmse6010024

**Value of the Data**

• Identified plastic items in river banks according to litter international classifications (MSFD and OSPAR) for comparisons with marine data.
• Reporting items in number, mass and volume for conversions between units in other studies dealing with plastic litter in rivers.
• The amount of plastic preproduction pellets is reported at levels never reported before.
• New types of items identified: fibers from toilet brushes, plastic tag ties, plastic fragments from road brushes.
• Need to adapt the OSPAR/MSFD classifications used for the marine environment to rivers.

## 1 Data

In this report, an inventory of plastic items is presented (Table 1). Plastic items were collected in a quadrat of 1 m^2^ in a historical polluted shore in the Seine river (downstream of the estuary; Lat. 49.4339; Long. 0.6160). Data are representative of the historical plastic pollution occurring in this river with few items dated from 1965, 1974, 1983, 1992 or 2010. Plastic items were classified according to OSPAR and MSFD classifications, which give insights about the origin of the items and their chemical composition.

**Table 1:** Inventory of plastic items collected in 1 m^2^ on a river bank in the Seine river at Petiville. In yellow, items of special interest for their high recurrence or their novelty.

A total of 20,259 plastic debris were individually counted, classified and weighted by category. Those plastic debris are more than 150% heavier in mass (> 4 kg) than organic debris, i.e. dead vegetation and gastropod shells, found in this kind of dry march surrounded by reedbeds.

**Fig. 1:**
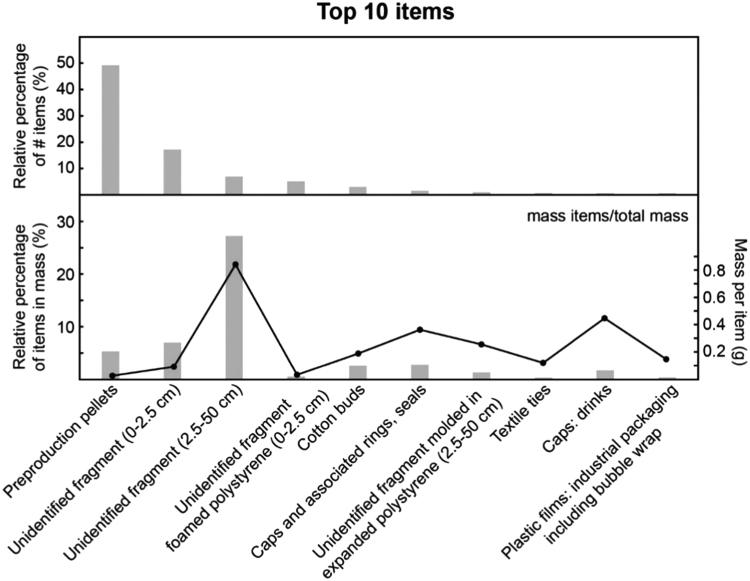
Top 10 plastic items collected in 1 m^2^ on a river bank in the Seine river at Petiville. The black line refers to the mass per unit

The Top 3 categories of items collected are plastic preproduction pellets [2], unidentified plastic fragments and plastic sticks (cotton bud and lollipop sticks; [1]. Plastic preproduction pellets are 15 times more numerous than gastropod shells. High concentration of pellets could be linked to the vicinity of plastic manufacturers near the sampled site. They represent 50% of the items collected during this campaign but only 5.6% of the mass (Fig. 1). In contrast, around 30% of the mass is carried by the unidentified fragments of macroplastics > 2.5 cm, which only represent 7% of the total items. Hundreds of caps, lids, and rings were also found without their associated bottles, which are often prompt to sink. Furthermore, the dataset refers to specific activities in the estuary with for example 100 g/m^2^ of polyethylene from shotgun plastic wads related to intense hunting activities. Those items have to be mentioned because they are very common in the estuary and their origin is clearly identified, while alternatives such as biodegradable wads do exist.

Reporting number of items, associated mass and volumes will improve conversions of unit for other studies related to river pollution when only one of the units are available. To facilitate conversions, mass per item were also reported for the Top 10 items (Fig. 1). In addition, specific items such as plastic tag ties (e.g. textile), or plastic fibers from toilet brushes were unusually reported and should be considered as additional categories in OSPAR/MSFD classifications for rivers.

## 2 Experimental design, materials, and methods

### 2.1 Experimental design

#### 2.1.1 Site description

**Fig. 2:**
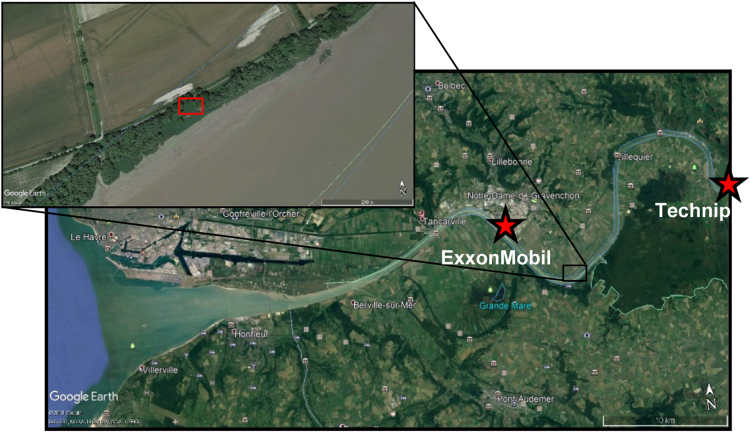
Geographical localization of the sampled site. Notice that plastic producers from Notre-Dame-de-Gravenchon are close to the targeted site. Lat. 49.4339; Long. 0.6160. The red stars point to the major plastic manufacturers, i.e. main sources of preproduction pellets.

Plastic litter were collected in the estuary of the Seine river close to Petiville, 80 km downstream of Rouen and 30 km upstream the river mouth (Fig. 2). Here is the beginning of the muddy plug of the estuary under high tidal influence. The sampled site belongs to a 1 km shore covered by plastic litter and corresponds to a visual maximum of plastic accumulation on a gentle slope dipping to the river. The depositional environment is a dried marsh annually flooded by the river and entrapped by a road on north-side (ancient towpath) and reedbeds on south-side. In this environment, woody debris and gastropod shells are very common. However, plastic debris also accumulate. They are not easily removed by flood events because reedbeds act as a barrier living floating items go in but not out. Very large items are less frequent thanks to this natural barrier and because of punctual cleanings by NGO׳s.

#### 2.1.2 Sampling method

Plastic litter and organics (wood and organisms, mostly dead gastropod shells) were exhaustively collected by hands in a quadrat of 1 m^2^ in a visual maximum of plastic accumulation until the soil was reached (see pictures in sup. data). Samples were stored in plastic bags of 50 L. They were dried at ambient air for days, then sorted and counted one by one in the lab, classified by size and category, and weighted.

#### 2.1.3 Classification method

Plastic items were classified according to OSPAR and MSFD classifications usually applied to marine environment and to macroplastics > 2.5 cm. Here, when possible, those classifications were also applied to items 0.5 cm < mesoplastics < 2.5 cm. They were separately numbered in the table but weighted together with macro-items. Only industrial pellets were numbered as microplastics (< 0.5 cm). Size class were determined based on at least one dimension.
