# Supplementary material for: Plastic debris dataset on the Seine river banks: Plastic pellets, unidentified plastic fragments and plastic sticks are the Top 3 items in a historical accumulation of plastics
Source: Data Brief. 2019 Jan 22;23:103697. doi: 10.1016/j.dib.2019.01.045 (PMC6369332; doi:10.1016/j.dib.2019.01.045)
Supplement: Supplementary file 1 — Supplementary material [file mmc1.docx]

All authors have no competing interests to declare
